# Chemopreventive Effect on Human Colon Adenocarcinoma Cells of Styrylquinolines: Synthesis, Cytotoxicity, Proapoptotic Effect and Molecular Docking Analysis

**DOI:** 10.3390/molecules27207108

**Published:** 2022-10-21

**Authors:** Vanesa Bedoya-Betancur, Elizabeth Correa, Juan Pablo Rendón, Andrés F. Yepes-Pérez, Wilson Cardona-Galeano, Tonny W. Naranjo

**Affiliations:** 1 Medical and Experimental Mycology Group, CIB-UPB-UdeA-UDES, Medellin 050034, Colombia; 2 Chemistry of Colombian Plants Group, Faculty of Exact and Natural Sciences, Institute of Chemistry, University of Antioquia (UdeA), Medellin 050010, Colombia; 3 School of Health Sciences, Pontificial Bolivarian University, Medellin 050034, Colombia

**Keywords:** styrylquinolines, colorectal cancer, antiproliferation, cell death, apoptosis, inflammation, reactive oxygen species, molecular docking

## Abstract

Seven styrylquinolines were synthesized in this study. Two of these styrylquinolines are new and were elucidated by spectroscopic analysis. The chemopreventive potential of these compounds was evaluated against SW480 human colon adenocarcinoma cells, its metastatic derivative SW620, and normal cells (HaCaT). According to the results, compounds **3a** and **3d** showed antiproliferative activity in SW480 and SW620 cells, but their effect seemed to be caused by different mechanisms of action. Compound **3a** induced apoptosis independent of ROS production, as evidenced by increased levels of caspase 3, and had an immunomodulatory effect, positively regulating the production of different immunological markers in malignant cell lines. In contrast, compound **3d** generated a pro-oxidant response and inhibited the growth of cancer cells, probably by another type of cell death other than apoptosis. Molecular docking studies indicated that the most active compound, **3a,** could efficiently bind to the proapoptotic human caspases-3 protein, a result that could provide valuable information on the biochemical mechanism for the in vitro cytotoxic response of this compound in SW620 colon carcinoma cell lines. The obtained results suggest that these compounds have chemopreventive potential against CRC, but more studies should be carried out to elucidate the molecular mechanisms of action of each of them in depth.

## 1. Introduction

According to the World Health Organization (WHO), cancer is one of the leading causes of death worldwide [1]. Colorectal cancer (CRC) was classified as the third most common type of cancer in the world and the second most frequent cause of death in 2020, presenting more than 1.9 million new cases and more than 900 thousand deaths [2].

Currently, different treatments are used for CRC, such as surgery, radiotherapy, chemotherapy, immunotherapy, and targeted therapy. However, the treatment of choice mainly depends on the stage of cancer and the general health of the patient. Among the most commonly used treatments in CRC, chemotherapy stands out as adjuvant therapy that is characterized by the administration of different drugs for the elimination of cancer cells, with 5-fluorouracil (5-FU) being one of the most frequently used in combination with other drugs (such as oxaliplatin, irinotecan, and leucovorin). However, these therapeutic options have been associated with multiple adverse effects, such as alopecia, gastrointestinal disorders, nausea, and vomiting, among other signs and symptoms that affect the quality of life of patients [3,4,5]. For this reason, the amount of research focused on cancer chemoprevention—defined as the use of natural, synthetic, or biological compounds to reduce the risk or delay the development of cancer—has increased in recent years.

One of the most studied natural compounds for cancer chemoprevention is resveratrol, a polyphenol belonging to the stilbene family that is found in grapes, peanuts, blackberries, and other foods of plant origin [6]. Antitumor, antioxidant, anti-inflammatory, cardioprotective and neuroprotective activities have been described for resveratrol, and they have been evidenced in both in vitro and in vivo assays [7,8,9]. Additionally, some studies have evaluated this compound in combination with 5-FU, demonstrating the ability of this stilbene to improve the effectiveness of 5-FU in CRC therapy [10]. Its antitumor effect has mainly been associated with its ability to modulate oxidative stress, inflammation, platelet aggregation, and the induction of tumor cell apoptosis [11,12,13]. On the other hand, 8-hydroxyquinoline is a heterocyclic organic compound known to be a chelating agent with antimicrobial and anticancer activity [14,15,16,17]. Specifically, its anticancer activity has been associated with its possible ability to inhibit the proliferation and migration of cancer cells through the induction of apoptosis and the generation of ROS, among other mechanisms [18,19,20].

**Figure 1:**
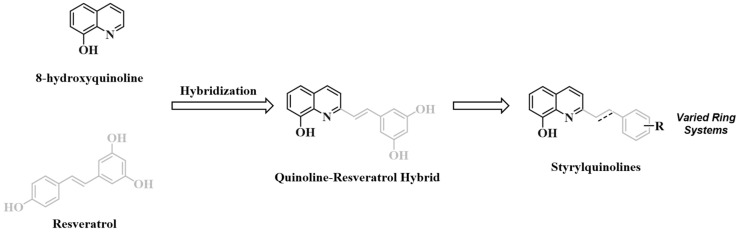
Design of styrylquinolines.

In recent years, the synthesis of hybrid compounds has gained importance for their use as therapeutic agents in different diseases, including cancer. These hybrid compounds have been defined as chemical entities comprising the partial or total structure of two or more molecules with different biological activities. This has allowed for the generation of new compounds that have shown greater safety, effectiveness at low doses, and tolerability to treatment due to improvements in pharmacokinetic properties and reductions in adverse effects related to the toxicity produced by the administration of multiple drugs [21,22]. An example of these hybrid compounds are the styrylquinolines, each comprising a quinoline nucleus attached to a styryl group (Figure 1), which have aroused significant interest in recent years given their potential as antiparasitic and antitumor agents [23,24,25]. However, there is not enough information about the mechanisms of action carried out by these hybrid compounds to exert their antitumor effect, specifically on colon cancer cells. On the other hand, there is no great evidence about their selectivity [25,26,27]. Thus, in order to find new molecules with antitumor potential, greater effectiveness and fewer side effects than currently available treatments for CRC, we evaluate the selectivity and antiproliferative capacity of hybrids based on 8-hydroxyquinoline (8-HQ) and resveratrol in vitro in human colon adenocarcinoma cells (SW480) and their metastatic derivative (SW620). Additionally, we determine the effect of these hybrids on different biological processes, such as apoptosis, the production of reactive oxygen species, and the regulation of different markers associated with inflammation. In this way, we will obtain preliminary results about the biological activity of these compounds that will be the basis for further research in more complex models, such as animal models.

## 2. Materials and Methods

### 2.1. Chemistry

#### 2.1.1. General Remarks

Microwave reactions were carried out in a CEM Discover (CEM, Matthews, NC, USA) microwave reactor in sealed vessels (monowave, maximum power of 300 W, temperature fixed with an IR sensor). ^1^H and ^13^C nuclear magnetic resonance (NMR) spectra were recorded on a Varian instrument (Palo alto, CA, USA) operating at 600 and 150 MHz. The signals of the deuterated solvent (CDCl_3_) were used as reference (CDCl_3_: δ = 7.27 ppm for ^1^H NMR and δ = 77.00 ppm for ^13^C NMR). Silica gel 60 (0.063–0.200 mesh, Merck, Whitehouse Station, NJ, USA) was used for column chromatography, and precoated silica gel plates (60 F254 0.2 mm, Merck, Whitehouse Station, NJ, USA) were used for thin-layer chromatography (TLC).

#### 2.1.2. General Procedure for the Synthesis of Styrylquinolines

##### Styrylquinolines **3a–e**

8-Hydroxyquinaldine (1 eq) and benzaldehyde (2 eq) were dissolved in acetic anhydride (20 mL) in a 50 mL flatbottomed flask equipped with a magnetic stirring bar. The mixture was stirred and heated to reflux under microwave irradiation for a period of 3 h. The crude reaction mixture was evaporated under reduced pressure, the residue was dissolved in methanol, and then 3 eq of KOH was added. This solution was stirred for 1 h. Finally, this solution was added to cold water in an ice bath. The resulting yellow solid was filtered, washed with water, and dried. The obtained solid was purified by column chromatography over silica gel eluting with mixtures of hexane and ethyl acetate of different ratios to obtain styrylquinolines with yields between 60% and 75%. The monitoring of the reaction progress and product purification was carried out by TLC.

##### Obtention of Compounds **4a** and **4b**

A solution of **3a** or **3d** (1 eq) in methanol was added under hydrogen to a suspension of Pd-C 10% (0.05%) in dry methanol (10 mL). The reaction was monitored by NMR until the consumption of the starting material. Filtration afforded compound **4a** at 90% and **4b** at 94%.

2-(4-hydroxyphenethyl)quinolin-8-ol (**4a**): ^1^H NMR (600 MHz, chloroform-d) δ 8.03 (d, *J* = 8.4 Hz, 1H), 7.39 (t_apparent_, *J* = 7.8 Hz, 1H), 7.29 (d, *J* = 8.0 Hz, 1H), 7.22 (d, *J* = 8.4 Hz, 1H), 7.15 (d, *J* = 7.8 Hz, 1H), 6.83 (d, *J* = 8.0 Hz, 1H), 6.75–6.67 (m, 2H), 3.81 (s, 3H), 3.26 (t, *J* = 7.8 Hz, 2H), 3.10 (t, *J* = 7.8 Hz, 2H). ^13^C NMR (151 MHz, Chloroform-d) δ 159.63, 151.76, 146.35, 143.84, 137.68, 136.19, 133.35, 126.87, 126.83, 122.49, 120.98, 117.60, 114.28, 111.10, 109.78, 55.85, 40.48, 35.09. EIMS: *m*/*z* 266.1292 [M + H]^+^, Calcd. for C_17_H_16_NO_2_: 266.1287.

2-(4-hydroxy-3-methoxyphenethyl)quinolin-8-ol (**4b**): ^1^H NMR (600 MHz, Chloroform-d) δ 8.02 (d, *J* = 8.4 Hz, 1H), 7.39 (t, *J* = 7.9 Hz, 1H), 7.28 (d, *J* = 8.4 Hz, 1H), 7.23 (d, *J* = 8.4 Hz, 1H), 7.15 (d, *J* = 7.8 Hz, 1H), 7.08 (d, *J* = 7.9 Hz, 2H), 6.75 (d, *J* = 7.8 Hz, 2H), 3.24 (t, *J* = 7.8 Hz, 2H), 3.10 (t, *J* = 7.8 Hz, 2H). ^13^C NMR (151 MHz, Chloroform-d) δ 159.63, 153.77, 151.74, 137.68, 136.19, 133.63, 129.57 (2C), 126.87, 126.81, 122.45, 117.61, 115.25 (2C), 109.77, 40.38, 34.49. EIMS: *m*/*z* 296.1395 [M + H]^+^, Calcd. for C_18_H_18_NO_3_: 296.1393.

### 2.2. Biological Activity Assays

#### 2.2.1. Cell Lines and Culture Conditions

For the biological assays, the human colon adenocarcinoma cell line SW480 and its metastatic derivative SW620 were used. Additionally, the nonmalignant cell line HaCaT was used to find the selectivity index of the compounds. All cell lines were obtained by the Colombian Plant Chemistry Group of the Faculty of Exact and Natural Sciences of the University of Antioquia (Medellín, Colombia) from the European Collection of Authenticated Cell Cultures (ECACC, Salisbury, UK). Cells were cultured in a DMEM medium with 4500 mg/L of glucose and L-glutamine (Sigma-Aldrich, Burlington, MA, USA), supplemented with 10% heat-inactivated (56 °C) horse serum (Gibco, Waltham, MA, USA), 1% penicillin/streptomycin (Sigma-Aldrich, Burlington, MA, USA), and 1% non-essential amino acids (Sigma-Aldrich, Burlington, MA, USA). For all experiments, horse serum was reduced to 3%, and the medium was supplemented with 5 mg/mL of transferrin, 5 ng/mL of selenium, and 10 mg/mL of insulin (ITS; Sigma-Aldrich, Burlington, MA, USA) [28]. All cell lines were incubated at 37 °C in a 5% CO_2_ atmosphere. Additionally, cell cultures were constantly monitored with PCR (Sigma-Aldrich, Burlington, MA, USA) for *Mycoplasma* spp. [29] to control contamination with this agent.

#### 2.2.2. Cytotoxic Activity

To evaluate the effect of styrylquinolines on the viability of the SW480, SW620, and HaCaT cell lines, sulforhodamine B (SRB) staining was used. SRB is a colorimetric assay that indirectly estimates the number of living cells based on the ability of SRB to bind to protein components of adherent cells [30]. Briefly, the malignant cell lines were seeded at a final density of 2.0 × 10^4^ cells/well, and the nonmalignant cell line was seeded at a final density of 1.5 × 10^4^ cells/well in 96-well tissue culture plates. All cell lines were incubated at 37 °C in a 5% CO_2_ atmosphere for 24 h to enable cell adherence, and they were then treated for 24 and 48 h with increasing concentrations (0.01–160 µM) of the styrylquinolines or their respective precursors (8-hydroxyquinoline and resveratrol), as well as 1% DMSO (negative control) and 5-FU (standard drug). After treatment, cells were fixed with trichloroacetic acid (50% *v*/*v*; PanReac AppliChem, Barcelona, Spain) for a period of one hour at 4 °C. After this, the cells were incubated for 30 min at room temperature with 0.4% (*w*/*v*) SRB (Sigma-Aldrich, Burlington, MA, USA). To remove unbound SRB, the cells were washed with 1% acetic acid, and the plates were allowed to dry at room temperature. Protein-bound SRB was solubilized with 10 mM Tris-base (Amresco, Cleveland, OH, USA) for 30 min at room temperature under constant agitation, and absorbance was measured at 490 nm in a microplate reader (Bio-Rad iMark^TM^, Hercules, CA, USA). Finally, the concentration of the compound that inhibits 50% of cell growth (IC50) and the selectivity index (SI) were determined.

#### 2.2.3. Antiproliferative Activity

The antiproliferative activity of styrylquinolines with higher selective cytotoxicity toward malignant cells was also tested with SRB staining [30]. Briefly, cells were seeded to a final density of 2.5 × 10^3^ cells/well in 96-well tissue culture plates and incubated under the same conditions described for cytotoxic activity. After 24 h, the cells were treated with increasing concentrations (5–80 µM) of the selected hybrids or with 1% DMSO (negative control) for 0, 2, 4, 6, and 8 days. Culture media were replaced every 48 h to guarantee the basic nutrients required for cell growth and viability, maintaining the concentrations of each of the selected hybrids. After each incubation time, cells were fixed and stained, and the absorbance was measured as described above.

#### 2.2.4. Reactive Oxygen Species (ROS) Levels

In order to evaluate the effect of the chosen styrylquinolines on the production of ROS in malignant cells, the 2′,7′-dichlorofluorescein diacetate (2′,7′-DCFDA) probe (Calbiochem, San Diego, CA, USA) was used as described by Kim et al. [31]. Briefly, malignant cells were seeded at a final density of 2.5 × 10^5^ cells/well in 6-well tissue culture plates for 24 h at 37 °C in a 5% CO_2_ atmosphere. Afterward, cells were treated for 24 and 48 h with 1% DMSO (negative control) or with the IC50 of the styrylquinolines obtained at 24 and 48 h of each cell line. After treatment, 2′,7′-DCFDA was added to a final concentration of 10 µM and incubated at 37 °C for 30 min. Finally, representative images of each well were taken using a fluorescence microscope (Axio Vert. A1; ZEISS, Jena, Germany) and Zen blue 3.4 software. The cell lysate was obtained from each well, and relative fluorescence units (RFU) were measured at excitation/emission wavelength (Ex/Em) = 485/525 nm using the Varioskan Lux microplate reader (Thermo Fisher Scientific, Waltham, MA, USA). The total protein concentration was quantified with the BCA method using the Pierce^TM^ BCA kit (Thermo Fisher Scientific, Waltham, MA, USA) to normalize the UFR.

#### 2.2.5. Assessment of Apoptosis

To assess whether the chosen styrylquinolines generated apoptosis in malignant cells, the APO-DIRECT^TM^ kit (Chemicon^R^ International, Temecula, CA, USA) was used following the manufacturer’s instructions. Briefly, the malignant cells were seeded at a final density of 1.1 × 10^6^ cells in a T75 culture flask and incubated for 24 h at 37 °C in a 5% CO_2_ atmosphere. Then, cells were treated for 24 and 48 h with 1% DMSO (negative control) or the IC50 obtained at 24 and 48 h for each compound. After incubation time, cells were fixed and stained with FITC-dUTP and propidium iodide (PI), respectively, according to the manufacturer’s protocol. Analysis was conducted via flow cytometry (LSR Fortessa; BD Biosciences, San Jose, CA, USA) and FlowJo 7.6 software. All PI-FITC-positive cells were considered to be apoptotic cells.

#### 2.2.6. Determination of Inflammatory Cytokines and Apoptotic Proteins

To assess whether styrylquinolines had any effect on the expression of immunological markers associated with the inflammatory process, malignant cells were seeded at a final density of 2.5 × 10^5^ cells/well in 6-well tissue culture plates, and cell adherence was allowed. After this, the cells were treated for 24 and 48 h with either 1% DMSO (negative control) or the IC50 obtained at 24 and 48 h of treatment with the compound that presented the best results in the aforementioned biological assays. After the incubation time, the supernatant was collected, and the levels of the following analytes were measured using the ProcartaPlex Human Th1/Th2/Th9/Th17/Th22/Treg 18-plex panel (Invitrogen) according to the manufacturer’s protocol: GM-CSF, IFN-γ, TNF-α, IL-10, IL-12p70, IL-13, IL-17A, IL-18, IL-1β, IL-2, IL-21, IL-22, IL-23, IL-27, IL-4, IL-5, IL-6, and IL-9. Furthermore, to evaluate the participation of these hybrids in the production of some markers associated with the apoptosis process (Bcl-2, active Caspase-3 and cleaved PARP) in colorectal cancer cells, the human apoptosis panel (Invitrogen, Waltham, Massachusetts, United States) was used following the manufacturer’s instructions. To normalize the concentration of each marker, total proteins were quantified using the PierceTM BCA kit (Thermo Fisher Scientific, Waltham, MA, USA).

In both cases, the reading was performed in the MAGPIX marker multiplex analyzer (Luminex XMAP, Austin, TX, USA). The concentration of each molecule was extrapolated from the calibration curve (individual for each marker) obtained from the standards provided by the kit.

#### 2.2.7. Statistical Analysis

All experiments were performed at least three times. The normality of the variables was evaluated using the Kolmogorov–Smirnov test. Data are expressed as the mean ± SE (standard error). IC50 values were evaluated by non-linear regression. Statistical differences between the negative control group (cells treated with 1% DMSO) and the treated cells at the different evaluation times were analyzed by two-way ANOVA followed by Dunnett’s test. Values with *p* ≤ 0.05 were considered significant. Data were analyzed using GraphPad Prism version 8 software for Windows (Graph Pad Software 8, San Diego, CA, USA).

### 2.3. Computational Methods

The 2D chemical structures of the most active styrylquinolines were drawn using ChemDraw 17.0 software (Cambridge Soft, Cambridge, MA, USA) and then saved as MDL MoL files. Chem3D 17.0 software (Cambridge Soft, Cambridge, MA, USA) was used to generate 3D structures of ligands, and optimization was performed using the MM2 Force-Field in Chem3D Ultra 8.0 Software CS, ChemOffice Chem3D Ultra 8.0, and Cambridge Soft. AutoDockTools (ADT) was used to parameterize ligands: non-polar hydrogens were merged, rotatable bonds were assigned, full hydrogens were added, and Kollman united partial atom charges were added to the individual protein atoms. The 3D protein structure of the caspase-3 (PDB ID: 5i9b) was downloaded from the Protein Data Bank website (accessed on 18 June 2022). Co-crystallized ligands, ions, and water molecules were removed from the protein structure by using DS Visualizer 2.5 program. For docking analysis, grid map dimensions (32 × 32 × 32 Å) were set surrounding the active site at x, y, and z coordinates of x = 1.5, y = −8.1, and z = −13.4 at an exhaustiveness of 20 for each protein–compound pair and a grid spacing of 1 Å. The AutoDock Vina v.1.2.0 software package by The Scripps Research Institute [32] was used with a flexible-ligand/rigid-receptor protocol and binding affinity/free energy estimated in kcal/mol. Finally, to inspect docking solutions, DS Visualizer 2.5 and PyMOL Molecular Graphics System Version 2.0 Schrodinger, LLC (2015) were used.

## 3. Results

### 3.1. Chemistry

**Scheme 1:**
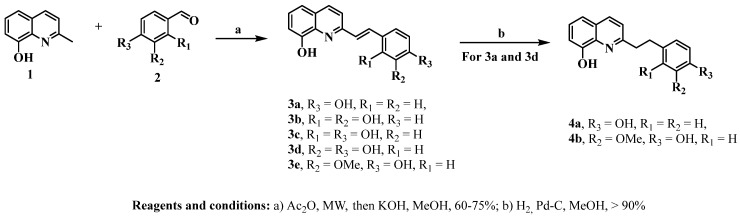
Synthetic pathway to the obtention of styrylquinolines.

Styrylquinolines **3a**–**e** were obtained via microwave-assisted, Perkin-type condensation between 8-hydroxyquinaldine (1) and benzaldehydes with different substituents (2) [33]. The reaction yields ranged between 60 and 75%. These compounds have already been reported [27,34,35,36,37]. However, our synthetic strategy involves microwave-assisted reactions that allow us to create compounds with shorter reaction times than those created using conventional heating methods. Here, the products were obtained in good-to-excellent yields and without appreciable by-product formation. Then, compounds **3a** and **3d** were reduced using catalytic hydrogenation and yielded **4a** and **4b**, respectively, at yields greater than 90% [38] (Scheme 1).

### 3.2. Effect of Styrylquinolines on Cell Viability of Malignant and Nonmalignant Cells

To determine the effect of hybrids based on resveratrol and 8-hydroxyquinoline on cell viability, different concentrations of styrylquinolines were evaluated in malignant cell lines (SW480 and SW620) and a nonmalignant cell line (HaCaT). Data are reported in terms of cytotoxicity, finding inhibitory concentration 50 (IC50), as shown in Table 1.

IC_50_ values were obtained from dose-response curves for each compound. The selectivity index (SI) was calculated as the ratio of IC_50_ values in nonmalignant HaCaT cells to the IC_50_ of SW480 cells or SW620 cells. Data are presented as the mean ± SE of at least three independent experiments. Compound 3e was not evaluated due to solubility problems.

After 24 h of treatment, it was observed that hybrid **3a** presented more selective cytotoxicity towards both malignant cell lines compared to the other styrylquinolines, as well as the precursors and the reference drug (5-FU). This was evidenced by the high IC50 values (231.9 ± 29.2 µM) in the non-malignant cell line (HaCaT) and the high SI values (SI_SW480-24h_ = 3.4; SI_SW620-24h_ = 3.8). In addition, hybrid **3c** also exhibited high selectivity on SW480 cells (SI = 3.0), and compound **3d** was selective towards the SW620 cell line (SI = 3.0). Although compound **3b** exhibited the lowest IC50 values among all tested hybrids (IC50 of 57.2 ± 6.3 µM, 72.6 ± 11.0 µM, and 49.1 ± 4.6 µM in HaCaT, SW480, and SW620 cells, respectively), its selectivity was significantly low because this hybrid also showed high cytotoxicity in the nonmalignant cell line. Similar results were obtained with hybrids **4a** and **4b**.

After 48 h of treatment, the SW620 cell line showed greater susceptibility to the evaluated hybrid compounds since it showed greater cytotoxicity in the metastatic line (IC50 of between 6.4 and 28.9 µM) compared to the SW480 cell line (IC50 of between 26.5 and 63.2 µM). Furthermore, hybrids **3a** and **3d** had the highest SI values (SI = 1.8 and 1.9, respectively) in the SW480 cell line. However, higher selectivities were observed in the SW620 cell line (SI ≥ 1.9), mainly for compound **3d** (SI = 12.3). 

On a structure-activity relationship basis, it was observed in SW480 cells that there was a decrease in activity in the presence of dihydroxylated compounds compared to the presence of monohydroxylated compounds (**3a** vs. **3b–d**). This relationship was not clear in SW620 cells. On the other hand, the presence of a double bond in a side chain is important for activity, i.e., a decrease in the effect was observed when the reduction was carried out (**3a** vs. **4a**). Similar results were obtained in other studies with cinnamic acid alkyl ester derivatives [39].

In accordance with the cytotoxicity and high SI, compounds **3a** and **3d** were chosen to continue with the other biological assays. The IC50 values obtained for each cell line at the different treatment times (24 and 48 h) were taken into account.

### 3.3. Antiproliferative Effect of Styrylquinolines

**Figure 2:**
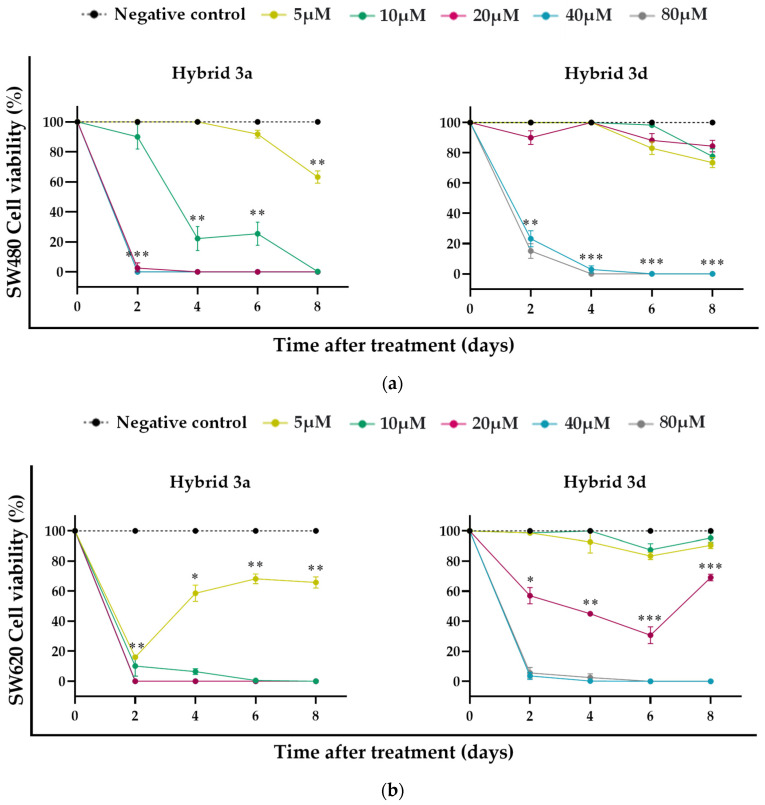
Antiproliferative effect of styrylquinolines **3a** and **3d** in the (**a**) SW480 and (**b**) SW620 cell lines. Data are presented as the mean ± SE of at least three replicates (* *p* < 0.05; ** *p* < 0.01; *** *p* < 0.001 vs. negative control). The negative control was assumed as 100% cell viability.

In order to evaluate the antiproliferative activity of compounds **3a** and **3d** on colorectal cancer cell lines, Sulforhodamine B (SRB) staining was used. As shown in Figure 2, hybrids **3a** and **3d** showed concentration- and time-dependent antiproliferative activity on malignant cells, as evidenced by the statistically significant decrease (*p* < 0.05) in the cell viability percentage compared to the negative control. Compound **3a** decreased the cell viability percentage to 0% using concentrations from 10 µM in both SW480 and SW620 cell lines. In contrast, compound **3d** required higher concentrations (fourfold more than those used with compound **3a**) to reach the same effect. This compound produces a high percentage of reduction in cell viability just from 40 µM concentration in both malignant cell lines.

The SW620 cells exhibited greater susceptibility to treatments compared to SW480 cells since the latter required higher concentrations (20 and 40 µM for **3a** and **3d**) to significantly decrease the cell viability percentage from day 2 post-treatment than did the metastatic cell line (5 and 20 µM for **3a** and **3d**, respectively) at the same time.

### 3.4. ROS Production Induced by Styrylquinolines

**Figure 3:**
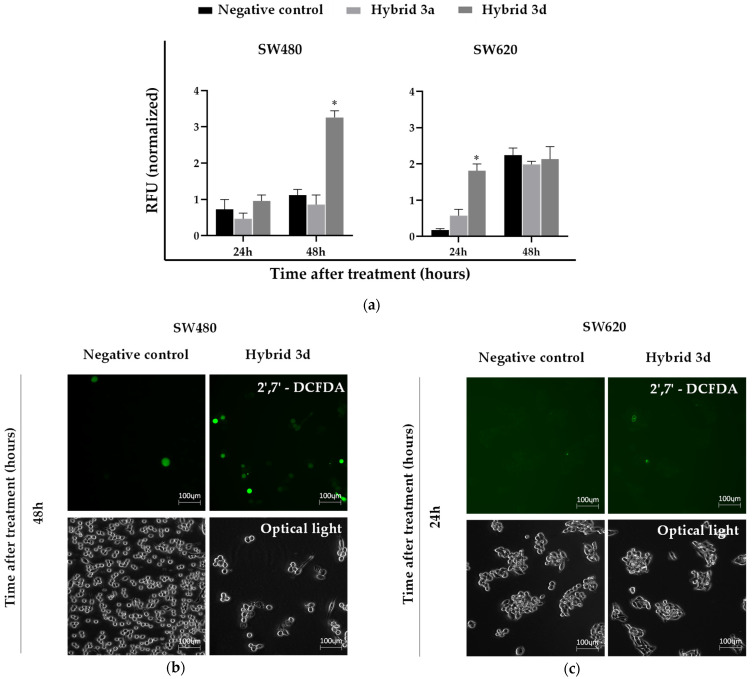
Intracellular ROS production: (**a**) quantification of ROS in SW480 and SW620 cell lines after 24 and 48 h of treatment with hybrids **3a** and **3d**; (**b**) representative images of the SW480 cell line at 48 h post-treatment with hybrid **3d** and (**c**) representative images of the SW620 cell line at 24 h post-treatment with hybrid **3d**. Magnification: 20×. Data are presented as the mean ± SE of at least three replicates (* *p* < 0.05 vs. negative control). RFU: Relative Fluorescence Units.

To assess the intracellular ROS production induced by hybrids **3a** and **3d** in colorectal cancer cell lines, a 2′,7′-DCFDA probe was used. According to the results, compound **3d** was the only one capable of inducing a statistically significant increase in ROS production compared to the control (*p* < 0.05; Figure 3a). The significant increase in ROS levels in the SW620 and SW480 cell lines occurred at 24 h (1.8 ± 0.1 RFU) and 48 h (3.3 ± 0.2 RFU) after treatment, respectively. The increase in ROS production by compound **3d** in the SW620 cell line was lower than in the SW480 cell line, which was evidenced by the low fluorescence observed in Figure 3c. Importantly, the cells showed morphological changes regarding size and shape after being treated (Figure 3b,c).

### 3.5. Apoptosis Induction by Styrylquinolines

**Figure 4:**
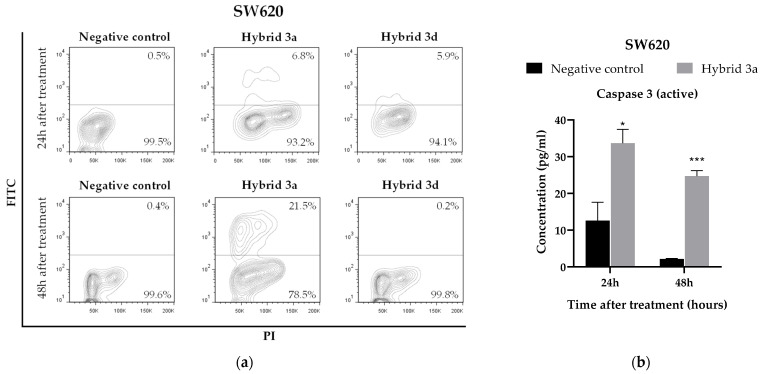
(**a**) Population of apoptotic cells of SW620 cell line treated with hybrids **3a** and **3d** for 24 and 48 h. (**b**) The concentration of markers associated with the apoptosis process in the SW620 cell line at 24 and 48 h after treatment with hybrid **3a**. Data are presented as the mean ± SE of at least three replicates (* *p* < 0.05; *** *p* < 0.001 vs. negative control). PI: Propidium Iodide.

In order to investigate whether the **3a** and **3d** hybrids caused apoptosis in CRC cell lines, a flow cytometry assay was performed using FITC-dUTP to label fragmented DNA. Hybrids **3a** and **3d** generated 6.8 and 5.9%, respectively, of cell death by apoptosis in the SW620 cell line after 24 h of treatment. Compound **3a** also generated an increase in apoptotic cells (21.5%) at 48 h post-treatment (Figure 4a). None of the hybrids generated apoptosis in the SW480 cell line (data not shown). 

Following the aforementioned results, the MAGPIX platform was used to evaluate whether compound **3a** regulated some markers (Bcl-2, active caspase 3 and cleaved PARP) involved in the cell death process in the SW620 cell line. Hybrid **3a** significantly increased the levels of active caspase 3 at both 24 and 48 h post-treatment in the metastatic cells (Figure 4b). The other evaluated markers (Bcl-2 and cleaved PARP) did not show significant differences when compared to the negative control.

### 3.6. Effect of Styrylquinolines on the Production of Immunological Markers Associated with the Inflammatory Response

**Figure 5:**
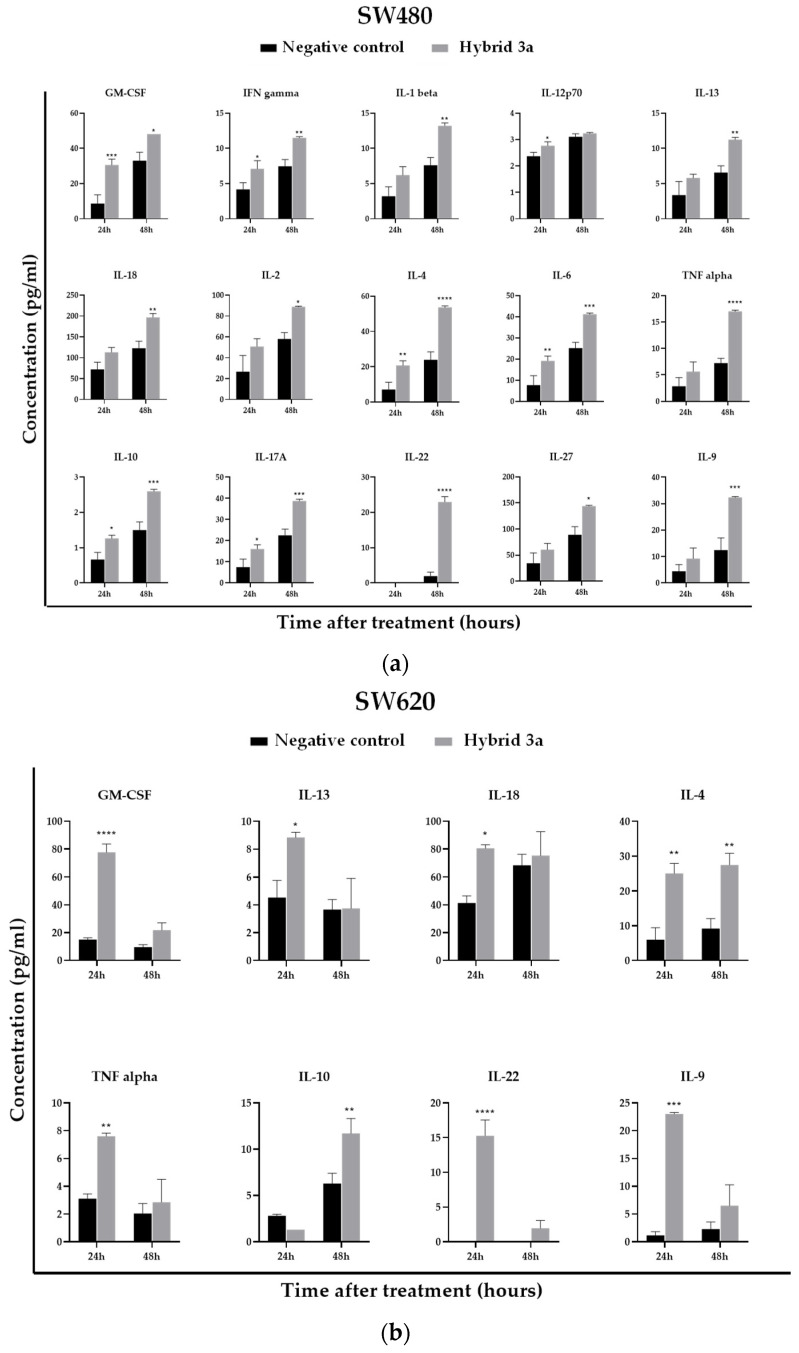
Levels of immunological markers associated with the inflammatory process in the (**a**) SW480 and (**b**) SW620 cell lines at 24 and 48 h post-treatment with hybrid **3a**. Data are presented as the mean ± SE of at least three replicates (* *p* < 0.05; ** *p* < 0.01; *** *p* < 0.001; **** *p* < 0.0001 vs. negative control).

To evaluate the immunomodulatory response of styrylquinolines in colorectal cancer cell lines, the hybrid with the best results obtained in the aforementioned biological assays was selected. For this purpose, the levels of some representative immunological markers of Th1 (GM-CSF, IFN-γ, TNF-α, IL-12p70, IL-1β, and IL-2), Th2 (IL-4, IL-5, IL-10, and IL-13), Th17/Treg (IL-17A, IL-6, IL-18, IL-21 and IL-23), Th22 (IL-22 and IL-27) and Th9 (IL-9) response were determined. As shown in Figure 5a, compound **3a** induced significant increases in the levels of GM-CSF, IFN gamma, IL-12p70, IL-4, IL-6, IL-10, and IL-17A in SW480 cells at 24 h post-treatment. Evidently, 48 h after treatment, a greater immunomodulatory effect was observed to maintain significant increases in GM-CSF, IFN gamma, IL-4, IL-6, IL-10 and IL-17A levels. Additionally, this hybrid induced significant increases in the levels of IL-1 beta, IL-13, IL-18, IL-2, TNF alpha, IL-22, IL-27, and IL-9 in this same post-treatment time.

Furthermore, hybrid **3a** induced a significant increase in the levels of GM-CSF, IL-13, IL-18, IL-4, TNF alpha, IL-22, and IL-9 at 24 h post-treatment in the SW620 cell line. At 48 h of treatment, the levels of IL-4 and IL-10 were significantly increased by this hybrid in the same cell line (see Figure 5b). The other evaluated cytokines did not show significant differences compared to the control group.

### 3.7. Molecular Docking Studies

According to biological assays, hybrid **3a** (4-hydroxy-styryl-substituted) caused a remarkable apoptotic effect in colorectal cancer cells. The in vitro cytotoxic response produced for **3a** in SW620 colon carcinoma cell lines appeared to be strongly associated with the modulation of caspase-3. Therefore, we hypothesized that **3a** targets caspase-3, thus altering its activity or function. In this scenario, we performed computational studies with the aim of exploring a possible binding mechanism of caspase-3 for compound **3a** using the docking program AutoDock Vina v.1.2.0. To accomplish this goal, compound **3a** was docked inside the catalytic domain of the X-ray crystallographic structures of caspase-3 (PDB code: 5i9b) protein, and their protein-ligand binding affinities (in kcal/mol) together with binding modes were estimated.

**Figure 6:**
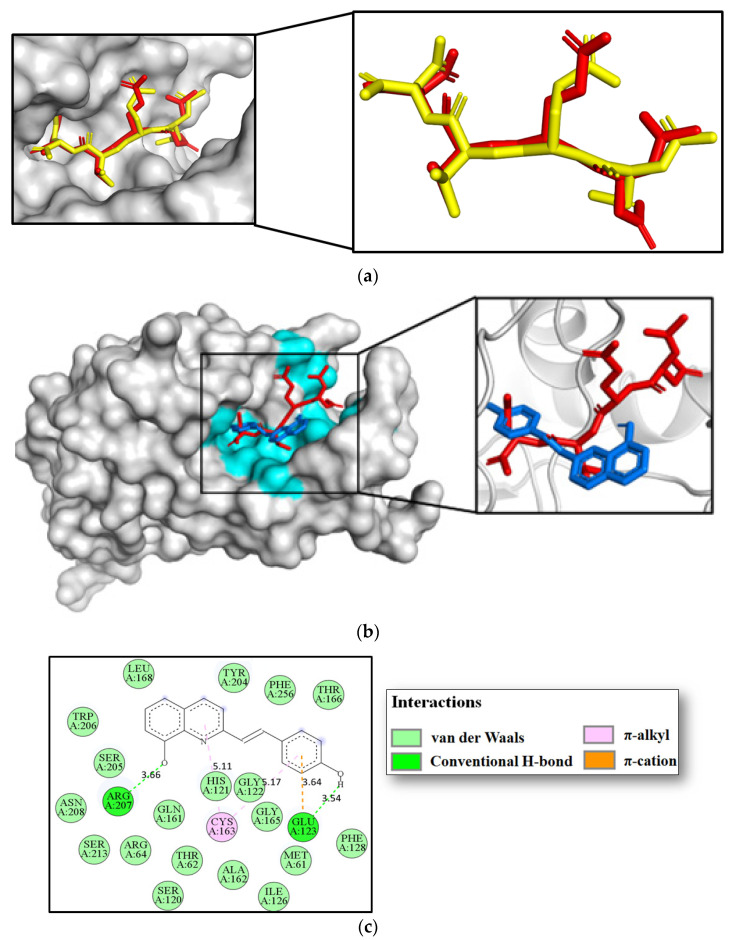
Docking studies of the **3a**-caspase-3 complex. (**a**) Self-docking study. 3D superposition of the best-docked pose of Ac-DEVD-CMK (in yellow) and its crystallographic binding pose (in red). (**b**) Alignment of the best-docked conformation of **3a** (in blue) and crystallographic binding mode of inhibitor Ac-DEVD-CMK (in red) within the caspase-3 catalytic domain. (**c**) 2D interaction diagram between **3a** and caspase-3. Hotspot amino acid residues are colored in cyan.

In our docking scheme, we first proceeded with self-docking simulations in order to validate our AutoDock Vina docking protocol. For this purpose, we carried out a comparison of the binding modes of the re-dock Ac-DEVD-CMK inhibitor (in yellow) and their crystallographic binding mode (in red) deposited in the PDB archive (PDB code: 5i9b) [40]. The results indicated that our docking procedure was able to reproduce the binding mode of the co-crystallized inhibitor Ac-DEVD-CMK (in red) with a strong root mean square deviation (RMSD) of 1.075 Å, showing a close homology (Figure 6a). This finding indicated a high level of feasibility in our protein-ligand docking protocol. After the docking procedure was validated, compound **3a** was docked into the caspase-3 catalytic domain. We found that hybrid **3a** (in blue) not only efficiently bound to caspase-3 with a closer binding affinity (−7.6 kcal/mol) than the current inhibitor Ac-DEVD-CMK (−8.2 kcal/mol) but it also fits well inside the catalytic cavity of caspase-3, as can be seen in Figure 6b. These facts support our experimental evidence suggesting that compound **3a** could prevent cell growth and proliferation in colorectal cancer cells by modulating caspase-3 function. Considering that the active site of caspase-3 comprises eighteen “hotspot” amino acid residues (Arg64, Leu119, Ser120, His121, Gln161, Ala162, Cys163, Ser198, Tyr204, Ser205, Trp206, Asn208, Ser209, Trp214, Ser249, Phe250, Ser251, and Phe252) [40], our modeling work also suggested that **3a** could bind to caspase-3 through several non-covalent interactions with those critical amino acid residues vital for caspase-3 function (Figure 6c) [40]. A close view of the 2D ligand-protein interaction plot after the docking procedure showed that **3a** interacted with the Arg207 residue via one hydrogen bond at a distance of 3.66 Å. Similarly, the styryl portion was found to create one hydrogen bond and one π–cation contact with the Glu123 residue at distances of 3.54 and 3.64 Å, respectively. We also noted that both the quinoline ring and the styryl moiety were able to bind to the caspase-3 via two π–alkyl contacts with Cys163. Figure 6c also shows numerous hydrophobic contacts that could play important roles in stabilizing the **3a**/caspase-3 complex following the binding event. These results suggest that two hydrogen bonding interactions, one π−cation and two π–alkyl contacts with those critical “hotspot” residues, could have important roles in the effective modulation of caspase-3 in **3a**-induced cytotoxicity.

## 4. Discussion

Previous studies have evaluated the antimicrobial activity of different styrylquinolines and antitumor activity in different cancer cell lines. However, more information is needed about the anticancer effect of these hybrids on CRC. In our study, the styrylquinoline compounds showed better activity in all cell lines than resveratrol and 5-Fu. Specifically, styrylquinolines **3a** and **3b** were more active than 8-HQ. These results show the importance of hybridization in the design of new drugs. Most of the evaluated hybrids showed a cytotoxic effect at low concentrations on the malignant cells compared with the reference drug, which only showed cytotoxic activity 48 h after treatment. These results are consistent with those of previous studies showing that other styrylquinolines also have cytotoxic activity at low concentrations (even < 10 µM) in different types of cancer cells [25,26]. It has been seen that 5-FU requires high concentrations to achieve a cytotoxic effect on different malignant cell lines, including SW480 and SW620 [30]. For this reason, many studies have focused on administering 5-FU in combination with other compounds to increase chemosensitivity in cancer cells and improve the effectiveness of this drug [41]. It should be noted that the chemosensitivity of cancer cells may be due to their heterogeneity, which may explain the variability in IC50 found in the literature for 5-FU in different cell lines and the different responses to the drugs used in cancer chemotherapy [42]. Even so, hybrids **3a** and **3d** were chosen to continue with the other biological assays because they showed greater selectivity towards malignant lines.

Regarding the antiproliferative activity in malignant colon cell lines, it was found that hybrids **3a** and **3d** inhibited cell proliferation in direct proportion to the time of treatment and to the concentration of each hybrid in cancer cells. Compound **3a** presented a significant inhibition of cell proliferation at lower concentrations than those required by compound **3d**, which may have been due to the difference in the number of functional groups and their positions in the structure of each hybrid, which provided them different properties and reactivity. Furthermore, greater cytotoxicity of the treatments was observed in the SW620 cell line, possibly because it has been described that SW480 and SW620 cells present differences in the karyotype and expression profile of microRNAs, which have been associated with CRC progression through regulation of some signaling pathways and with chemoresistance to some drugs [43,44,45,46]. In the same sense, it has been seen that certain post-translational modifications of some cellular proteins have been gaining importance in the study of many diseases, including different types of cancer [47]. Many studies have investigated the role of different proteins susceptible to glycosylation and have described their association with resistance to some drugs used in cancer chemotherapy [48,49]. In this way, the differences in the response of the cell lines used in our study to the treatment with the hybrids evaluated could be explained.

In order to study the mechanisms by which these hybrids exert an antiproliferative effect on colon cancer cells, the levels of ROS production, apoptosis, and some immunological markers associated with inflammation were evaluated. ROS production occurs as a consequence of the normal physiological aerobic metabolism of a cell [50]. The production of ROS occurs as a consequence of the normal physiological aerobic metabolism of a cell, but when there is an imbalance between the production of ROS and the antioxidant mechanisms used by the cell to remove ROS, an accumulation of these free radicals occurs within the cell and causes damage to lipids, proteins and DNA; this process has been associated with the development of various diseases, including cancer [51]. In parallel, the overproduction of ROS in cancer cells induced by different compounds has been associated with cell death, which is why some current therapeutic strategies are focused on evaluating this phenomenon as an antitumor mechanism [52,53]. The results obtained in this study showed that compound **3d** had a pro-oxidant effect on SW620 and SW480 cells but did not induce apoptosis in either cell line. This effect of styrylquinolines may be due to the chelating properties of 8-hydroxyquinoline, one of the precursors of these hybrid compounds since this chelation process can lead to the formation of ROS and cause oxidative damage to cells [27,54]. It is important to highlight that ROS overproduction has also been associated with other types of cell death that are not characterized by nuclear fragmentation, such as ferroptosis and necroptosis [55,56,57]. A study by Lee SH. and Lee YJ. showed that resveratrol, in combination with docetaxel (a drug used to treat different types of cancer), concurrently induced apoptosis and necroptosis in prostate carcinoma cells [58]. In another study conducted by Lee J. et al., it was shown that resveratrol increased ferroptosis in head and neck cancer cells through the induction of the activation of the protein sirtuin 1, which has been associated with increased susceptibility to this type of cell death [59]. Thus, it is possible that the mechanism used by compound **3d** to induce cell death in colorectal cancer cells was something other than apoptosis.

It is known that apoptosis is a type of programmed cell death that can occur in two ways depending on the stimulus that triggers it. The intrinsic or mitochondrial pathway is activated by multiple factors that generate cellular stress, e.g., DNA damage, the increased production of free oxygen radicals, and endoplasmic reticulum stress. On the other hand, the extrinsic pathway is activated by the binding of different ligands to death receptors expressed in the cell, such as Fas and TRAIL receptors (TNF-related apoptosis-inducing ligand) [60]. Our results showed that compound **3a** induced apoptosis in SW620 cells and generated a significant increase in caspase 3 levels in this same cell line. Moreover, molecular docking analysis showed that compound **3a** effectively bound with caspase 3 protein, obtaining a comparable binding affinity (−7.6 kcal/mol) to that of the Ac-DEVD-CMK inhibitor. Therefore, combined experimental and computational findings indicated that the modulation of this protein might be a possible molecular mechanism to understand the cytotoxic response of **3a** in the SW620 colon carcinoma cell line. However, determining the pathway by which apoptosis is carried out requires further evaluation because caspase 3 is one of the effector proteins shared by both pathways of apoptosis (intrinsic and extrinsic) [61].

Compound **3a** presented the best results throughout this study, which is why it was chosen to evaluate its effect on the regulation of different immunological markers in CRC cells. There is significant evidence of an association between increases in the levels of certain cytokines related to the inflammatory process and the progression of cancer. However, cytokines have pleiotropic properties that enable them to play a dual role in cancer. They can participate in pro- or anti-tumor responses depending on the stage and microenvironment of the tumor [62]. Thus, in vitro and in vivo studies and some clinical trials have shown an association between increases in the levels of some cytokines and antitumor responses. Some studies have demonstrated that IL-2 can induce T cell proliferation and differentiation, as well as cause its activation [63]. A study by Ding et al. demonstrated the ability of IL-27 to enhance T cell anti-tumor immunity enhancing cell survival and memory T cell differentiation [64]. Another cytokine that has been associated with an antitumor response is IL-9, which favors the activation of cytotoxic T lymphocytes by recruiting dendritic cells to tumor tissues for the presentation of these antigens and the subsequent elimination of malignant cells [65,66]. Although the role of IL-22 in cancer has not been well-elucidated and it is controversial, it has been described that it has a positive prognosis in this type of cancer, relying on its capacity to induce a cross-talk between tumor cells and immune cells associated with a favorable clinical outcome [67]. IL-4 seems to have an important role in Th9 cell priming and differentiation, which have been associated with a powerful antitumor capacity [68]. IL-13 promotes the migration of dendritic cells and the activation of cytotoxic T lymphocytes [69]. In this same sense, Chen et al. demonstrated that IL-17 has the ability to modulate neutrophil-mediated antitumor immunity in cells [70]. IL-6 seems to promote antitumor immunity mediated by a Th17 response [71,72], and IFN-γ promotes tumor antigen presentation [73]. GM-CSF production seems to have a synergistic effect with Toll-Like Receptor—2 (TLR2) to inhibit tumor growth and modulate tumor-infiltrating Antigen Presenting Cells (APCs) [74], and IL-18 has been associated with better survival rates in cancer patients [75]. Finally, our results showed that compound **3a** upregulated different immunological markers such as GM-CSF, IFN gamma, IL-4, IL-6, IL-10, IL-17A, IL-1 beta, IL-13, IL-18, IL-2, TNF alpha, IL-22, IL-27, and IL-9, indicating that its antitumor effect is probably caused by its high immunomodulatory capacity.

In conclusion, the findings obtained in this study suggest that hybrids **3a** and **3d** have chemopreventive potential against CRC. Both compounds inhibited the proliferation of SW480 human colon adenocarcinoma cells and their metastatic derivative SW620. Compound **3a** was shown to be more effective at lower concentrations than those required by compound **3d**. The mechanisms by which these compounds exert their antiproliferative effect on malignant cell lines appear to be different. Hybrid **3d** promoted SW480 and SW620 cell death, probably through another mechanism different from apoptosis that may be related to ROS production. Hybrid **3a** induced apoptosis in SW620 cells, as evidenced by nuclear fragmentation and increased levels of active caspase 3 in these cells. Additionally, hybrid **3a** exhibited a high immunomodulatory effect, upregulating most of the immunological markers evaluated in this study. However, further experimental and computational studies are needed to clearly delineate the cytotoxic mechanism associated with styrilquinoline **3a**, preferably in other more complex models, in order to assess its effect on the immune response in a tumor microenvironment.

## Figures and Tables

**Table 1:** Cytotoxic effect (IC50) of styrylquinolines on SW480, SW620 and HaCaT cell lines at 24 and 48 h post-treatment.

Compound	24 h					48 h				
	HaCaT IC_50_ (µM)	SW480 IC_50_ (µM)	SI	SW620 IC_50_ (µM)	SI	HaCaT IC_50_ (µM)	SW480 IC_50_ (µM)	SI	SW620 IC_50_ (µM)	SI
3a	231.9 ± 29.2	67.7 ± 2.8	3.4	60.5 ± 2.9	3.8	47.9 ± 3.3	26.5 ± 4.0	1.8	16.6 ± 2.2	2.9
3b	57.2 ± 6.3	72.6 ± 11.0	0.8	49.1 ± 4.6	1.2	33.5 ± 4.9	39.0 ± 3.6	0.9	9.7 ± 2.0	3.4
3c	110.5 ± 12.7	37.1 ± 3.8	3.0	62.0 ± 4.0	1.8	38.4 ± 6.2	38.4 ± 3.8	1.0	20.7 ± 3.1	1.9
3d	163.9 ± 14.1	159.7 ± 16.6	1.0	54.6 ± 7.5	3.0	78.9 ± 2.3	42.5 ± 5.2	1.9	6.4 ± 1.1	12.3
4a	65.1 ± 8.4	117.4 ± 5.3	0.6	51.0 ± 5.6	1.3	69.7 ± 3.0	63.2 ± 7.1	1.1	28.9 ± 1.2	2.4
4b	109.3 ± 8.1	93.4 ± 16.6	1.2	58.8 ± 5.5	1.9	53.6 ± 2.2	43.2 ± 2.0	1.2	18.6 ± 2.0	2.9
Resveratrol	179.9 ± 7.2	217.1 ± 12.8	0.8	168.9 ± 3.9	1.1	98.2 ± 7.0	135.7 ± 9.1	0.7	125.2 ± 4.6	0.8
8-HQ	87.1 ± 9.4	89.6 ± 11.8	1.0	55.3 ± 3.6	1.6	61.6 ± 4.8	38.8 ± 3.2	1.6	36.3 ± 4.8	1.7
5-FU	>2000	>2000	2.0	>2000	0.9	38.78 ± 7.2	748.3 ± 157.6	0.4	295.5 ± 39.7	0.1

IC50 values were obtained from dose-response curves for each compound. The selectivity index (SI) was calculated by the ratio of IC50 values in nonmalignant cells (HaCaT) to the IC50 of malignant cells (SW480 or SW620). Data are presented as the mean ± SE of at least three independent experiments. Compound 3e was not evaluated due to solubility problems. 8-HQ: 8-hydroxyquinoline; 5-FU: 5-Fluorouracil.

## Data Availability

Not applicable.

## Sample Availability

Samples of the compounds are available from the authors.
